# Voluntary Medical Male Circumcision for HIV Prevention in Malawi: Modeling the Impact and Cost of Focusing the Program by Client Age and Geography

**DOI:** 10.1371/journal.pone.0156521

**Published:** 2016-07-13

**Authors:** Katharine Kripke, Frank Chimbwandira, Zebedee Mwandi, Faustin Matchere, Melissa Schnure, Jason Reed, Delivette Castor, Sema Sgaier, Emmanuel Njeuhmeli

**Affiliations:** 1 Health Policy Project, Avenir Health, Washington, DC, United States of America; 2 Ministry of Health, Lilongwe, Malawi; 3 United States Agency for International Development (USAID), Lilongwe, Malawi; 4 United States Department of Defense, Lilongwe, Malawi; 5 Health Policy Project, Futures Group, Washington, DC, United States of America; 6 USAID, Washington, DC, United States of America; 7 Bill & Melinda Gates Foundation, Seattle, Washington, United States of America; 8 University of Washington, Seattle, Washington, United States of America; University of Pittsburgh, UNITED STATES

## Abstract

**Background:**

In 2007, the World Health Organization (WHO) recommended scaling up voluntary medical male circumcision (VMMC) in priority countries with high HIV prevalence and low male circumcision (MC) prevalence. According to the Joint United Nations Programme on HIV/AIDS (UNAIDS), an estimated 5.8 million males had undergone VMMC by the end of 2013. Implementation experience has raised questions about the need to refocus VMMC programs on specific subpopulations for the greatest epidemiological impact and programmatic effectiveness. As Malawi prepared its national operational plan for VMMC, it sought to examine the impacts of focusing on specific subpopulations by age and region.

**Methods:**

We used the Decision Makers’ Program Planning Toolkit, Version 2.0, to study the impact of scaling up VMMC to different target populations of Malawi. National MC prevalence by age group from the 2010 Demographic and Health Survey was scaled according to the MC prevalence for each district and then halved, to adjust for over-reporting of circumcision. In-country stakeholders advised a VMMC unit cost of $100, based on implementation experience. We derived a cost of $451 per patient-year for antiretroviral therapy from costs collected as part of a strategic planning exercise previously conducted in- country by UNAIDS.

**Results:**

Over a fifteen-year period, circumcising males ages 10–29 would avert 75% of HIV infections, and circumcising males ages 10–34 would avert 88% of infections, compared to the current strategy of circumcising males ages 15–49. The Ministry of Health’s South West and South East health zones had the lowest cost per HIV infection averted. Moreover, VMMC met WHO’s definition of cost-effectiveness (that is, the cost per disability-adjusted life-year [DALY] saved was less than three times the per capita gross domestic product) in all health zones except Central East. Comparing urban versus rural areas in the country, we found that circumcising men in urban areas would be both cost-effective and cost-saving, with a VMMC cost per DALY saved of $120 USD and with 15 years of VMMC implementation resulting in lifetime HIV treatment costs savings of $331 million USD.

**Conclusions:**

Based on the age analyses and programmatic experience, Malawi’s VMMC operational plan focuses on males ages 10–34 in all districts in the South East and South West zones, as well as Lilongwe (an urban district in the Central zone). This plan covers 14 of the 28 districts in the country.

## Introduction

Voluntary medical male circumcision (VMMC) has been shown to reduce the sexual transmission of HIV from women to men by about 60% [1–3] and is a recommended HIV prevention strategy in countries with high HIV prevalence and low levels of male circumcision [4]. Malawi is one of 14 countries in Eastern and Southern Africa scaling up VMMC for HIV prevention following the recommendations of the World Health Organization (WHO) and the Joint United Nations Programme on HIV/AIDS (UNAIDS) [5]. The VMMC program in Malawi began in late 2011 and was formally launched in 2012 along with the national VMMC policy [6]. Although VMMC is identified as a priority HIV prevention intervention in key HIV policy documents including the National Strategic Plan for HIV and AIDS (2015–2020) [7] and the National HIV Prevention Strategy 2015–2020 [8], the Voluntary Medical Male Circumcision Strategy and National Operations Plan for Scale Up 2015–2020 is Malawi’s first VMMC strategy and operational plan [6].

Modeling studies were conducted in 2009–2011 [9] and informed the UNAIDS/WHO Joint Strategic Action Framework (JSAF) [5], which called for rapid expansion of VMMC services in 14 countries, in line with universal access objectives for all HIV interventions. Results showed that in Malawi, achieving 80% circumcision prevalence among males ages 15–49 within five years and maintaining this coverage level in subsequent years could avert an estimated 270,000 new HIV infections within 15 years and save more than $1 billion USD in treatment and care [5,9]. (All subsequent references to currency are in U.S. dollars.) The number of VMMCs required to reach 80% coverage in five years in Malawi was estimated at that time to be 2.1 million [9].

Despite efforts to scale up VMMC, the cumulative number of VMMCs performed through 2014 in Malawi since the introduction of VMMC in the country in 2011 was 150,000 [6]—considerably less than needed to reach 80% coverage. In addition to delays in policy adoption and planning around VMMC, implementation has faced a number of challenges both on the supply and demand sides [6]. On the supply side, constraints have included healthcare worker capacity to perform VMMCs and the amount of time that trained staff are able to dedicate to VMMC, leading to variable subpopulation coverage. Other supply-side challenges are limited funding and few implementing partners. On the demand side, uptake of VMMC varies considerably by age. Generally, adults do not favor VMMC or appreciate its health benefits; efforts to generate demand for circumcision have had limited success [10]. This is evident in routine program data showing that fewer than 10% of VMMC clients reached were 30- to 49-year-olds, even though this age group constitutes more than 25% of the potential clients (uncircumcised males ages 10–49). Conversely, the program data show that almost 30% of VMMC clients reached were 10- to 14-year-olds. This significant variation by age calls into question the practicality and impact of focusing on all 15- to 49-year-old males, rather than focusing on a specific age range. Ongoing modeling work by different groups on age targeting of VMMC interventions suggests that cost-effectiveness and VMMC impact can vary significantly across target age groups and HIV settings [11–13].

In addition, as is the case in other countries [14,15], in Malawi evidence of significant heterogeneity in HIV prevalence and incidence by age and across geographic areas is growing [16]. According to the most recent national household survey (2010) measuring HIV prevalence, important differences exist between rural and urban areas, with HIV prevalence estimated at 8.9% in rural areas and 17.4% in urban areas [16]. HIV prevalence also varies by region: At 14.5%, HIV prevalence in the Southern Region is about twice as high as in the Central (7.6%) and Northern (6.6%) regions [16].

Incorporating this new information, the Decision Makers’ Program Planning Toolkit, Version 2.0 (DMPPT 2.0) [17], was applied in 2013–2014 to explore the impact and cost-effectiveness of age and geographic targeting of VMMC scale-up in Malawi, to inform the development of the country’s Voluntary Medical Male Circumcision Strategy and National Operations Plan for Scale Up 2015–2020. This modeling exercise built on the previous VMMC modeling done for Malawi in 2011. The earlier effort applied the original version of the DMPPT to assess the cost and impact of national and uniform scale-up to 80% circumcision prevalence within five years with no variation by age or geographic location, and with males ages 15–49 as the target population [9].

## Methods

### Overview of DMPPT 2.0

The DMPPT 2.0 model is described in detail elsewhere [17]. Briefly, it is a simple compartmental model implemented in Microsoft Excel 2010 that can be used to analyze the effects of age at circumcision on program impact and cost. The model tracks the number of circumcised males among newborns and in each five-year age group over time, taking into account age progression and mortality. The model does not track circumcisions by HIV status; the target population is all uncircumcised men, regardless of HIV status. The model calculates discounted VMMC program costs and HIV infections averted in the population in each year across user-specified VMMC scale-up scenarios, compared with a baseline scenario in which the male circumcision (MC) prevalence is not increased above levels predating initiation of the VMMC for HIV prevention program (no scale-up). For Malawi, costs, numbers of circumcisions, and infections averted were all discounted at a rate of 3% per year.

### Data used

The DMPPT 2.0 model is populated with population, mortality, and HIV incidence and prevalence projections from an external source. For Malawi’s HIV incidence, we used a single Spectrum/AIM file in which the surveillance sites had been categorized by zone, and the incidence was calculated for each zone. HIV incidence by age from a national Spectrum/Goals model [18,19] for Malawi (S1 Fig, showing incidence by year for the total population, ages 15–49) was multiplied by the ratio of the HIV incidence in each zone (exported from the Spectrum/AIM file) to the national incidence from the Spectrum/AIM file to produce the age-specific HIV incidence for each zone, as shown in the equation below:

$$I_{a,t,z}=I_{a,t,Goals}*\left(\frac{I_{t,Zone}}{I_{t,Nat}}\right)$$

where *I*_*a*,*t*,*z*_ is the HIV incidence for a given age, year, and zone; *I*_*a*,*t*,*Goals*_ is the national HIV incidence for a given age and year from Goals; *I*_*t*,*Zone*_ is the HIV incidence across ages 15–49 in a given year in a given zone from the Spectrum/AIM model; and *I*_*t*,*Nat*_ is the national HIV incidence across ages 15–49 in a given year from the Spectrum/AIM model.

Population by age and year, mortality by age and year, and annual number of male births were exported from this Spectrum/Goals file into a national Malawi DMPPT 2.0 file.

The Malawi Ministry of Health has divided the country into five “health zones” for program management purposes: Central East, Central West, Northern, South East, and South West. The modeling team was requested to conduct the geographic analysis at the level of these health zones. For the zonal analyses, we used five Spectrum/AIM [20] files corresponding to the five health zones, which had previously been validated by the country. A separate DMPPT 2.0 file was created for each zone. Population, mortality, and births were exported from the zonal Spectrum/AIM file. For the incidence, we used a single national file in which the surveillance sites had been categorized by zone, and estimated HIV incidence for each zone using the R-Spline method in AIM. Surveillance data for this file included sentinel surveillance data through 2010 as well as survey data from the 2004 and 2010 Demographic and Health Surveys. We then multiplied HIV incidence by age from the Goals file by the ratio of the HIV incidence in a given zone (exported from the Spectrum/AIM file) to the national HIV incidence for each year between 2013 and 2020. In the Spectrum/Goals file, all HIV interventions were held constant in future years starting with 2012 coverage levels. For antiretroviral therapy (ART), coverage from 2013 and on was 63% among males and 74% among females, with treatment eligibility set for patients with CD4 counts below 350. For the years from 2021–2050, the zonal to national incidence ratio for 2020 was used to scale the national incidence by age.

We handled the urban/rural analyses in the same manner as the zonal analyses, except that we did not have individual validated Spectrum/AIM files for the rural and urban populations, so exporting population, deaths, and births by urban and rural categories was not possible. Population, deaths, and births for the DMPPT 2.0 models were calculated by multiplying the national population, deaths, and births by the urban to national or rural to national population ratios for each year (exported from the Spectrum/AIM urban/rural file). The incidence was handled in the same manner as described above for the zonal estimates.

Implementing partners of the U.S. President’s Emergency Plan for AIDS Relief (PEPFAR) compiled the numbers of VMMCs conducted in the country in each district between September 2012 and October 2013 from their program records and provided this information, disaggregated by five-year age group, on November 12, 2013. Male circumcision (MC) prevalence by age group in the model base year (2014) was derived from the 2010 Malawi Demographic and Health Survey [16]. The 2010 MC prevalence was reported at 2.5%, 10.1%, and 37.8% in the Northern, Central, and Southern regions, respectively [16]. These estimates were scaled according to the MC prevalence for each district [10] and then halved, at the request of country stakeholders, to adjust for over-reporting of circumcision [21]. The model assumed a unit cost of VMMC of $100, based on an expenditure analysis conducted by the PEPFAR team in Malawi. The annual per-person cost of ART was assumed to be $451, covering first- and second-line antiretroviral drugs, laboratory costs, tuberculosis and cotrimazole prophylaxis, and service delivery costs, based on ART costs validated by country stakeholders.

### Scenarios analyzed

For all scenarios, the baseline year was 2014 and the observation period was 2015–2029 inclusive, with two distinct periods: a scale-up phase from 2015–2019 and a maintenance phase from 2020–2029. VMMC coverage was increased from 2014 levels to target coverage levels by 2019 and maintained at target coverage levels thereafter. All scenarios assume a linear increase in coverage from 2015–2019.

For comparison, we also modeled a scenario (hereafter, the “reference scenario”) reflecting the country’s initial VMMC target, which was to increase circumcision coverage to 80% among males ages 15–49. Anticipating constraints on resources and other limitations, stakeholders in Malawi wished to explore the epidemiological and economic impacts of scaling circumcision to 60% rather than 80% (within five years). The other scenarios therefore scaled up VMMC coverage to 60% of males in the following age groups: 15–49 years, 10–49 years, 10–34 years, and 10–29 years. The 10–14 year age group was added to some of the scenarios, because VMMC uptake is high in this age group, as noted in the introduction to this paper. Similarly, the 30–49 and the 35–49 year age groups were omitted in select scenarios, because VMMC acceptability is relatively lower among these age groups.

To assess variation in the impact and cost of scaling up VMMC coverage in different parts of Malawi, two approaches were applied. First, given the geographic variation in HIV prevalence in Malawi [16], we looked at scenarios in which VMMC coverage was scaled up in each of Malawi’s five health zones. For each health zone, male circumcision coverage was scaled up to 60% of males ages 10–34. Second, because of the differences in HIV prevalence in rural and urban areas in Malawi [16], we created urban vs. rural scale-up scenarios where circumcision coverage was scaled up to 60% among males ages 10–34 in both the urban and rural Malawi DMPPT 2.0 models.

### Definitions of “cost-effective” and “cost saving”

Cost-effectiveness was expressed in terms of the cost per HIV infection averted by providing VMMC to the indicated population over a 15-year period (2015–2029), compared with a reference scenario in which VMMC is not scaled up over baseline levels. Cost-effectiveness was also expressed in terms of the incremental cost-effectiveness ratio (ICER), which is the cost per disability-adjusted life-year (DALY) saved. The cost per DALY saved was calculated by dividing the cost per HIV infection averted over the period of 2015–2029 by 20 [22]. Following WHO criteria, a specific VMMC scale-up scenario is considered cost-effective if the cost per DALY saved is less than three times the per capita gross domestic product (GDP) [23]. (The 2012 GDP per capita for Malawi—$268—was obtained from the World Bank [24].)

A VMMC scale-up scenario was considered cost-saving if the cost of the program from 2015–2029 was less than the lifetime cost of providing ART to the number of people whose infections were averted in that VMMC scale-up scenario over that same period. The lifetime cost of providing ART was estimated to be $6,450, with the annual cost starting eight years following HIV infection and taking into account annual discounting at a rate of 3%, as well as a 1% annual mortality rate on ART and non-AIDS mortality exported from Spectrum.

## Results

Malawi conducted a complete analysis of impact, cost, and cost-effectiveness of VMMC by client age, as outlined in [17] (see S2–S4 Figs). The impact and cost-effectiveness of VMMC scale-up for the five age scenarios that policymakers wished to compare are presented in Table 1. Under the reference scenario of scaling up VMMC to 80% coverage of males ages 15–49, almost 148,000 HIV infections would be averted. First, by aligning with the stakeholders’ choice of 60% rather than 80% coverage (but maintaining the target age group of 15- to 49-year-olds), the estimated number of HIV infections averted decreased to roughly 104,000.

**Table 1:** Impact and cost of VMMC scale-up by target age group, 2015–2029.

Target age group	Target VMMC coverage (%)	HIV infections averted (thousands)	VMMCs per HIV infection averted	Cost per HIV infection averted (thousands, USD)	Treatment costs averted (millions, USD)	ICER (cost per DALY saved)	Cost savings (millions, USD)
10–29	60	79	51	$5.1	$715	$268	$313
10–34	60	92	47	$4.6	$776	$232	$344
10–49	60	106	46	$4.6	$821	$223	$336
15–49	60	104	36	$3.6	$932	$176	$561
15–49	80	148	35	$3.5	$1,266	$177	$744

ICER: incremental cost-effectiveness ratio; DALY: disability-adjusted life year

Next, we explored the impacts of adding younger age groups into this target and removing the older age groups (while maintaining a goal of 60% coverage). Adding 10- to 14-year-olds to this target (making the target 10- to 49-year-olds, at 60% coverage) averted almost 2,000 additional HIV infections, or 102% of the infections averted in the scenario in which MC coverage is scaled up to 60% among 15- to 49-year-olds (Table 1). Removing males ages 35–49 (making the target 10- to 34-year-olds, at 60% coverage) averted roughly 92,000 HIV infections, or 88% of the infections averted in the scenario in which MC coverage is scaled up to 60% among 15- to 49-year-olds. Further narrowing the target to males ages 10–29 (removing males ages 30–49, and maintaining 60% coverage) averted about 79,000 HIV infections, or 75% of the infections averted in the scenario in which MC coverage is scaled up to 60% among 15- to 49-year-olds.

Whereas the cost per HIV infection averted was $5,127 in the scenario focusing scale-up on males ages 10–29, it was $4,638 when scale-up focused on males ages 10–34. Moreover, although focusing scale-up on either age group would be cost saving, focusing on males ages 10–34 increased net savings by $31 million as compared to circumcising 10- to 29- year-olds, assuming that the cost per VMMC is the same across all age groups.

**Fig 1:**
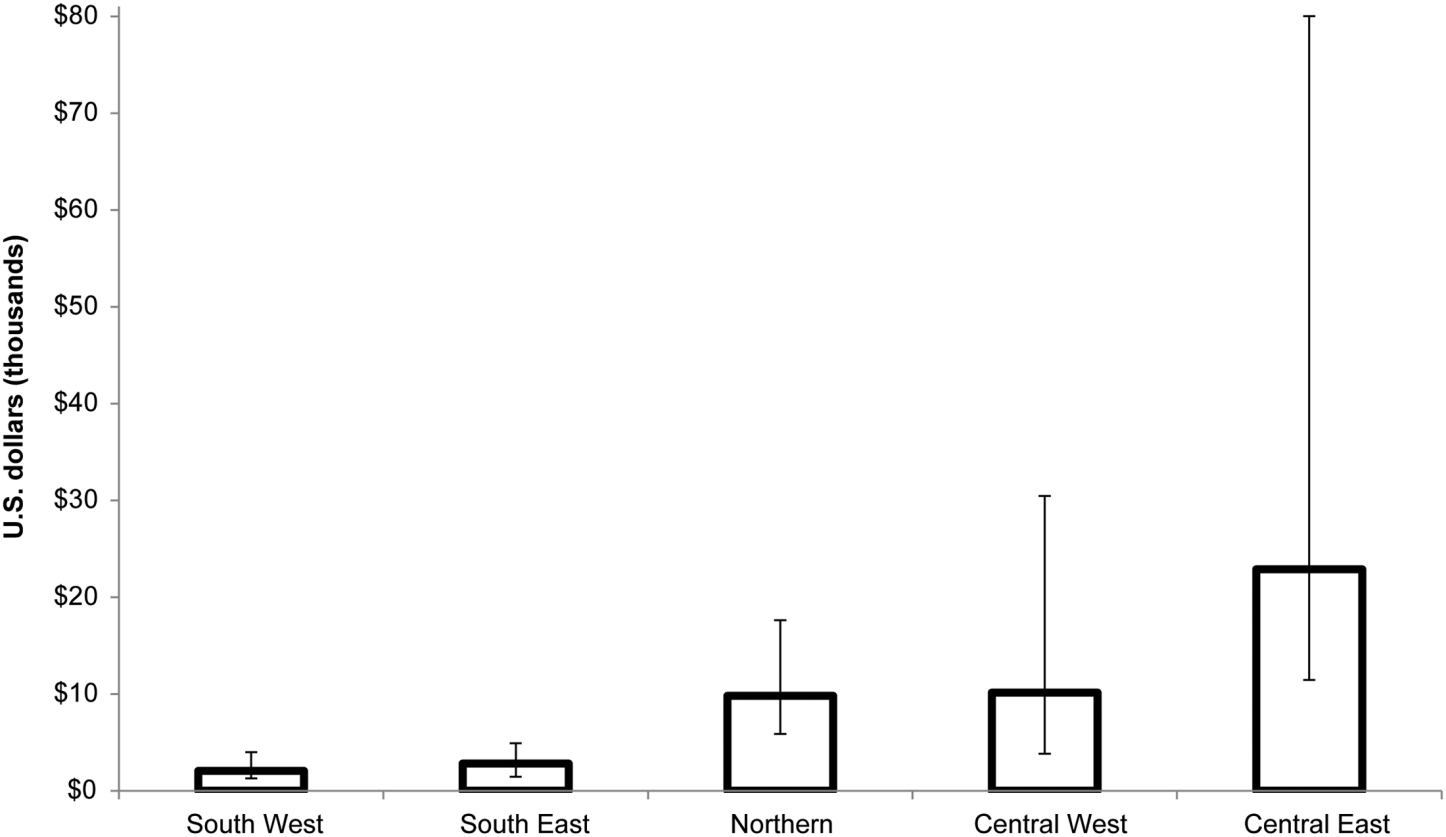
Discounted cost per HIV infection averted, by zone, 2015–2029. Error bars represent lower and upper uncertainty bounds as described in [17].

Because Malawi’s HIV epidemic is geographically diverse, policymakers wished to assess the impact and cost-effectiveness of focusing on different geographic areas. Table 2 presents the impact and cost-effectiveness of scaling up VMMC coverage to 60% among males ages 10–34 in each of the five health zones in Malawi between 2015 and 2029. The largest number of HIV infections averted was found in the South West and South East zones. These zones also had the lowest number of VMMCs required to avert one HIV infection: 19 and 25 VMMCs per infection averted, respectively. As a result, these zones also had the lowest cost per HIV infection averted, as Fig 1 illustrates. This remained true when we took the uncertainty bounds around the estimates into account. VMMC was cost-effective in all health zones except Central East. In the South West and South East zones, scaling up coverage to 60% of males ages 10–34 was also cost saving (Table 2).

**Table 2:** Impact and cost of scaling-up VMMC coverage to 60% among males ages 10–34, by health zone, 2015–2029.

Health zone	HIV infections averted (thousands)	VMMCs per HIV infection averted	Cost per HIV infection averted (thousands, USD)	Treatment costs averted (millions, USD)	ICER (Cost per DALY saved, USD)	Cost-savings (millions, USD)
Central East	3.4	207	$22.8	$26	$1,143	-$51
Central West	1.3	92	$10.2	$93	$508	-$34
Northern	7.0	89	$9.8	$53	$490	-$15
South East	32	25	$2.8	$225	$140	$139
South West	38	19	$2.0	$268	$103	$192

The HIV incidence estimates underlying the impact and cost-effectiveness results by zone are based on HIV surveillance data across the entire zone, and therefore they do not reflect differences in HIV incidence between urban and rural areas. Lilongwe, for example, is a major urban center in the Central West Zone, where the VMMC program was found to be less cost-effective than in the two southern zones. We hypothesized that the VMMC program in urban centers would be both cost-effective and cost-saving. There are insufficient HIV surveillance data to produce HIV incidence estimates for each zone disaggregated by urban and rural areas. Therefore, we looked at the results obtained using HIV incidence estimates from urban and rural areas across the country. Table 3 presents the impact and cost-effectiveness of scaling up VMMC coverage to 60% of males ages 10–34 in urban and rural Malawi. Scaling up VMMC coverage in urban areas would avert more than 51,000 HIV infections; an equivalent scale-up in rural areas would avert about 44,000 HIV infections. The number of VMMCs per HIV infection averted between 2015 and 2029 was 22 in urban areas and more than 60 in rural areas. VMMC was cost-effective and cost-saving both in urban and rural areas, although the cost per HIV infection averted was substantially lower and the cost savings were substantially higher in urban areas.

**Table 3:** Impact and cost of scaling up VMMC coverage to 60% among males ages 10–34 in urban and rural Malawi, 2015–2029.

Geographic area	HIV infections averted (thousands)	VMMCs per HIV infection averted	Cost per HIV infection averted (thousands, USD)	Treatment costs averted (millions, USD)	ICER (cost per DALY saved, USD)	Cost-savings (millions. USD)
Urban	51	22	$2.4	$455	$120	$331
Rural	44	64	$7.1	$388	$355	$74

## Limitations

The limitations of the DMPPT 2.0 model have been described elsewhere [17]. The following limitations should also be considered when interpreting these findings.

The estimate of VMMC’s unit cost was based on PEPFAR expenditure data, not data from a facility-based costing study. We assumed that the VMMC unit cost would be the same regardless of client age, but in fact it is probable that the unit cost varies by client age, given that demand creation efforts (and associated costs) would need to increase to cover recruitment of men over age 25. (The exact variation of VMMC unit cost by client age is currently unknown, but data may be obtained in the future from ongoing evaluation of VMMC demand creation strategies and the role of incentives at the collective and individual level, implemented by the Malawi National AIDS Commission and the Ministry of Health with World Bank support.) We also assumed that the VMMC unit cost did not vary by urban/rural location or by zone, although the cost would be expected to vary by geographic area based on different models of implementation: for example, mobile, outreach, and fixed sites; the use of salaried vs. temporary staff, and so forth. Costs may also change if Malawi introduces the PrePex^™^ device in its VMMC program.

As previously mentioned, HIV surveillance data are insufficient to produce HIV incidence estimates for each zone disaggregated by urban and rural areas. Therefore, we looked at the results obtained using HIV incidence estimates from urban and rural areas across the country. This assumption limits the strength of the modeled projections by urban and rural areas, because the projections are highly sensitive to estimates of HIV incidence, especially when extended over longer periods.

This analysis also assumes no migration in or out of the country or between health zones. Policies related to geographic targeting of VMMC services should take migration into account.

This modeling exercise provides insight into the impact and cost of varying the target age groups and geographic focuses of Malawi’s VMMC program, but it does not provide information on the “how” of scaling up VMMC in Malawi. It does not address the programmatic considerations critical to scale-up, including the demand creation necessary and its cost or the human resource requirements—for example, the importance of task-shifting for providers in rural health centers.

## Discussion

Recognition is growing that response to the HIV epidemic needs to move from broad national-level plans to a sharper focus on the populations and geographic areas at highest risk of acquiring the virus, for limited resources to have the greatest impact [11,12,19,25–27]. The modeling analyses detailed in this paper illustrate that the potential impact and cost-effectiveness of VMMC scale-up in Malawi are not uniform. They vary by the age group of males circumcised and also across geographic areas. VMMC scale-up is most cost-effective and has the biggest impact in the South East and South West zones and in urban areas.

This modeling exercise provided evidence to inform Malawi’s Voluntary Medical Male Circumcision Strategy and National Operations Plan for Scale Up 2015–2020, which proposes new VMMC scale-up plans for the country. In response to this evidence, the country strategy is now to focus on scaling up VMMC to 60% coverage among males ages 10–34 in 14 of the 28 districts. These 14 districts are Lilongwe (in the Central Zone) and all 13 districts comprising the South East and South West zones. Scaling up coverage to 60% of males ages 10–34 in the rest of the country is planned to occur by 2025.

A key contribution of this modeling analysis is the demonstration that changing the VMMC scale-up target to males ages 10–34 (adding males ages 10–14 and removing males ages 35–49) averts 88% of the HIV infections that would be averted by pursuing the 15- to 49-year-olds scenario. Given the difficulties in recruiting males ages 35–49 years, the HIV infections averted by circumcising this age group would not be worth the additional effort and cost required to recruit them. Of course, though the current country strategy identifies the implementation priorities, it should be noted that males ages 35–49 will not be turned away from services.

The country chose age 10 as the lower bound for scenarios to consider, because a large proportion of current VMMC clients are 10–14 years old and because establishing this threshold reduces the most new infections over the long term (beyond 2029). This was done even though, over the time frame studied, inclusion of males ages 10–14 leads to only small increases in HIV infections averted and results in a higher cost per HIV infection averted. Although an analysis focused on short-term impact might lead one to conclude that circumcising males ages 10–14 should not be a program priority, turning away medically eligible circumcision clients is viewed as problematic by implementers, given the limited demand for VMMC in Malawi to date. There is also the risk that some of those who are turned away may end up getting circumcised in traditional settings, where the risk of adverse events and the likelihood of incomplete removal of the foreskin are high [10].

By conducting the DMPPT 2.0 application and making direct use of its results in the VMMC strategy and operations plan for 2015–2020, the Government of Malawi has demonstrated its commitment to prioritizing the scale-up of VMMC for HIV prevention. These actions are in line with the recommendations of WHO and UNAIDS, and are testimony to the government’s desire to improve the lives of the people of Malawi.

## Supporting Information

S1 Fig Projected HIV incidence from Malawi Spectrum/Goals model. This figure shows the HIV incidence from the Malawi Spectrum/Goals model, projected to 2050.
(TIF)

S2 Fig Immediacy of impact: incidence rate reductions by age-specific scale-up strategy, 2014–2049. This figure depicts the reduction in HIV incidence for each age-group scenario, relative to a scenario with no scale-up, over the period of 2014–2049. Each age-group scenario here assumes that circumcisions are only performed in the specified age group, with effects being measured across the entire population. For each scenario, male circumcision is scaled up to 60% coverage. An incidence ratio of 1 would indicate that HIV incidence has not been affected by the circumcisions performed. This figure illustrates that the greatest short-term reduction in incidence (over the scale-up period of 2015–2019, arrow a) results from circumcising the age groups 20–24, 25–29, and 30–34, while circumcising age groups 15–19 and 20–24 provides the greatest reduction after 15 years (2015–2029, arrow b).
(TIF)

S3 Fig Magnitude of impact: total HIV infections averted by age-specific scale-up strategy, 2015–2029. This figure illustrates the magnitude of impact, or the total number of HIV infections averted by each age strategy. In contrast with the previous figure, S3 Fig depicts more realistic scenarios, in which circumcisions are not limited to specific five-year age groups but are scaled up to 60% coverage across wider combined age groups. The most impactful scenario would be one in which most clients are circumcised: the 10- to 49-year-olds scenario. Error bars represent lower and upper uncertainty bounds as described in [17].
(TIF)

S4 Fig Cost per HIV infection averted by age-specific scale-up strategy, 2015–2029. This figure compares the cost per HIV infection averted in each indicated scenario. As in S3 Fig, the scenarios compared here are combined age groups, and they again involve scaling up circumcision coverage to 60% of males in each indicated age group. Circumcising 60% of 15- to 49-year-olds would result in a cost of $3,500 per each infection averted. The lowest cost per HIV infection averted is achieved by circumcising males ages 15–34. Error bars represent lower and upper uncertainty bounds as described in [17].
(TIF)
