# Correction: Voluntary Medical Male Circumcision for HIV Prevention in Malawi: Modeling the Impact and Cost of Focusing the Program by Client Age and Geography

**DOI:** 10.1371/journal.pone.0169708

**Published:** 2017-01-03

**Authors:** Katharine Kripke, Frank Chimbwandira, Zebedee Mwandi, Faustin Matchere, Melissa Schnure, Jason Reed, Delivette Castor, Sema Sgaier, Emmanuel Njeuhmeli

There is an error in the Funding statement. The number for the Cooperative Agreement Project SOAR (Supporting Operational AIDS Research) is incorrect. The correct number is OAA-A-14-00060.
