# Supplementary figures and images for: Voluntary Medical Male Circumcision for HIV Prevention in Malawi: Modeling the Impact and Cost of Focusing the Program by Client Age and Geography
Source: PLoS One. 2016 Jul 13;11(7):e0156521. doi: 10.1371/journal.pone.0156521 (PMC4943664; doi:10.1371/journal.pone.0156521)

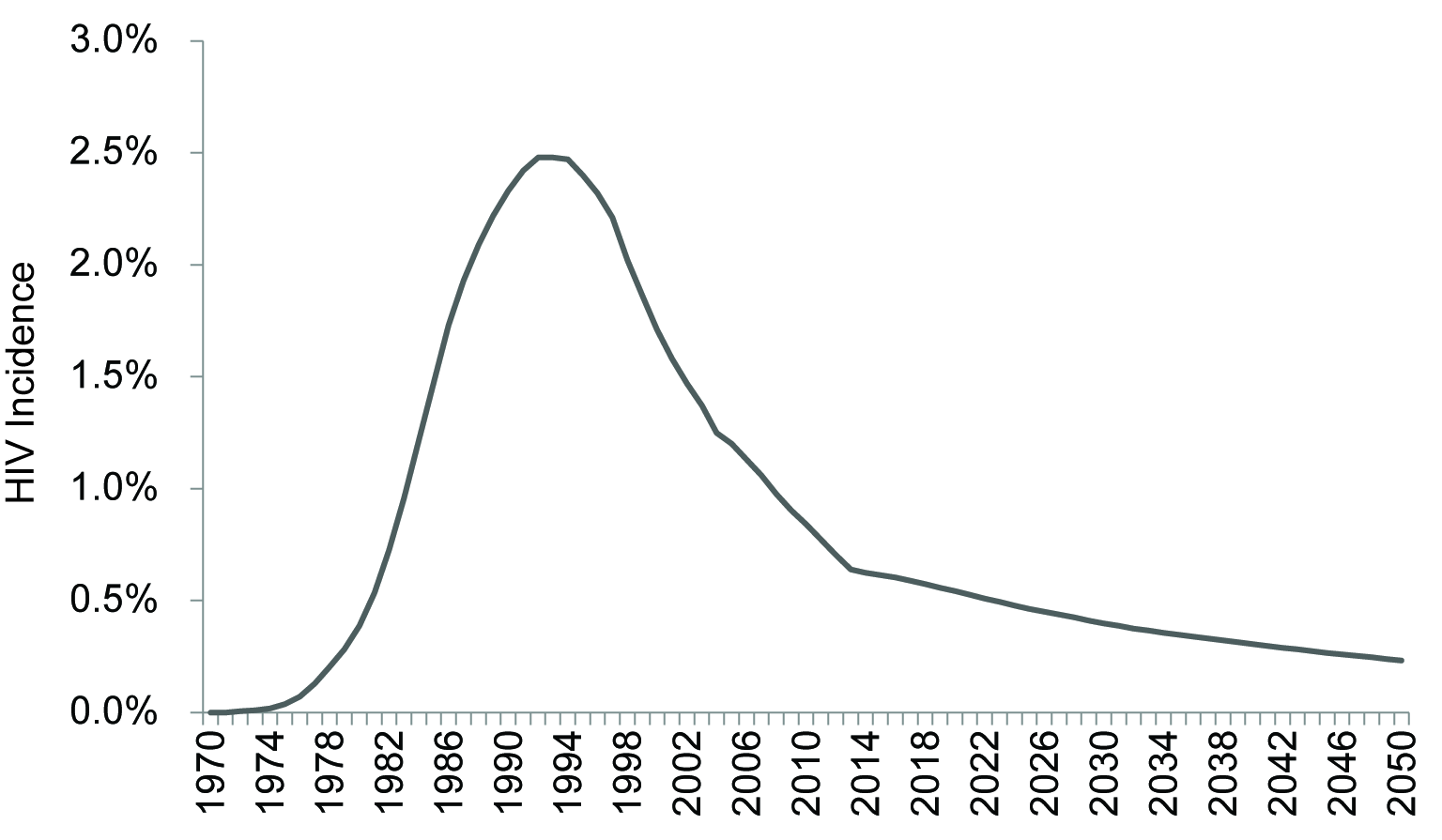

Supplement: S1 Fig — This figure shows the HIV incidence from the Malawi Spectrum/Goals model, projected to 2050. (TIF) [file pone.0156521.s001.tif]

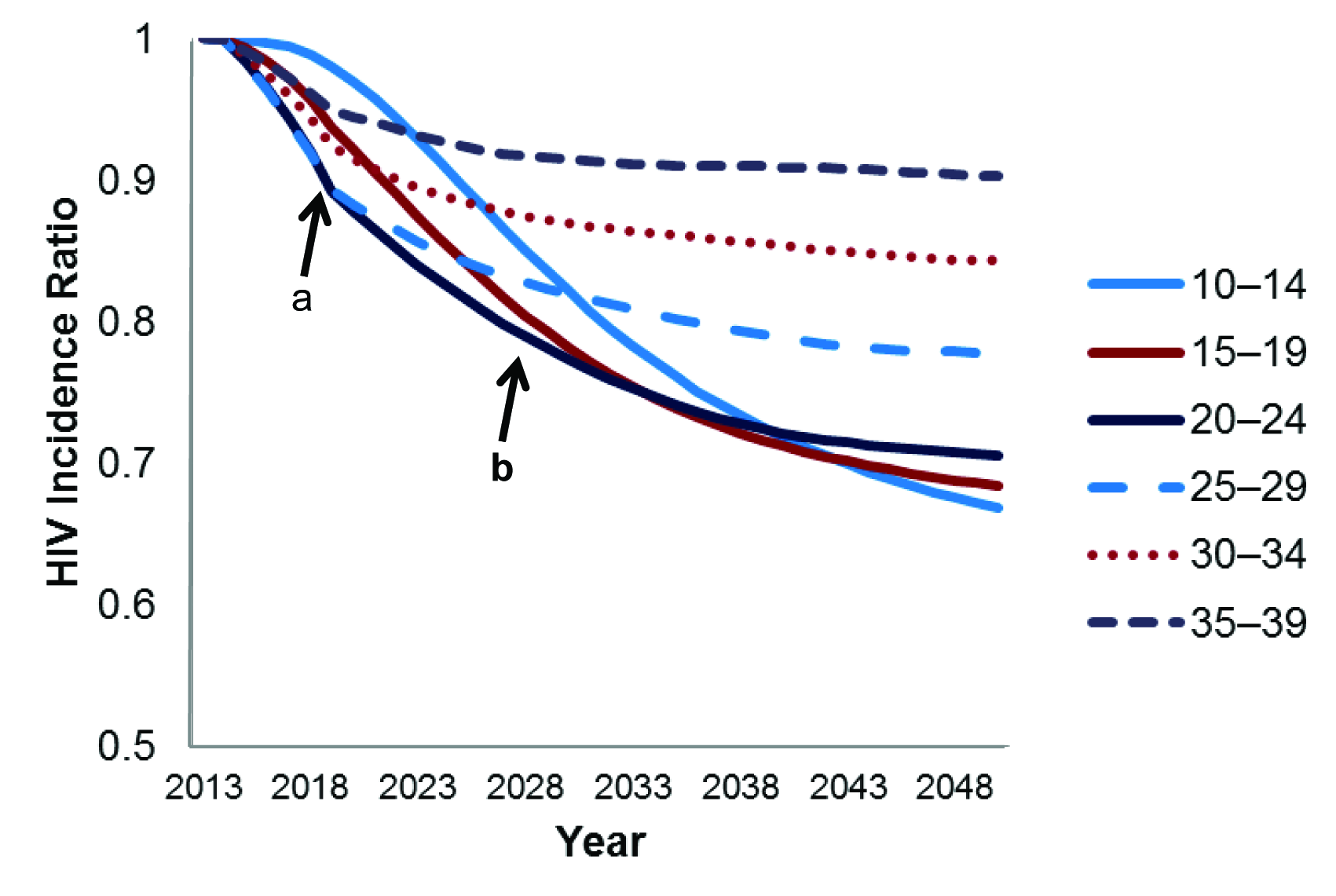

Supplement: S2 Fig — This figure depicts the reduction in HIV incidence for each age-group scenario, relative to a scenario with no scale-up, over the period of 2014–2049. Each age-group scenario here assumes that circumcisions are only performed in the specified age group, with effects being measured across the entire population. For each scenario, male circumcision is scaled up to 60% coverage. An incidence ratio of 1 would indicate that HIV incidence has not been affected by the circumcisions performed. This figure illustrates that the greatest short-term reduction in incidence (over the scale-up period of 2015–2019, arrow a) results from circumcising the age groups 20–24, 25–29, and 30–34, while circumcising age groups 15–19 and 20–24 provides the greatest reduction after 15 years (2015–2029, arrow b). (TIF) [file pone.0156521.s002.tif]

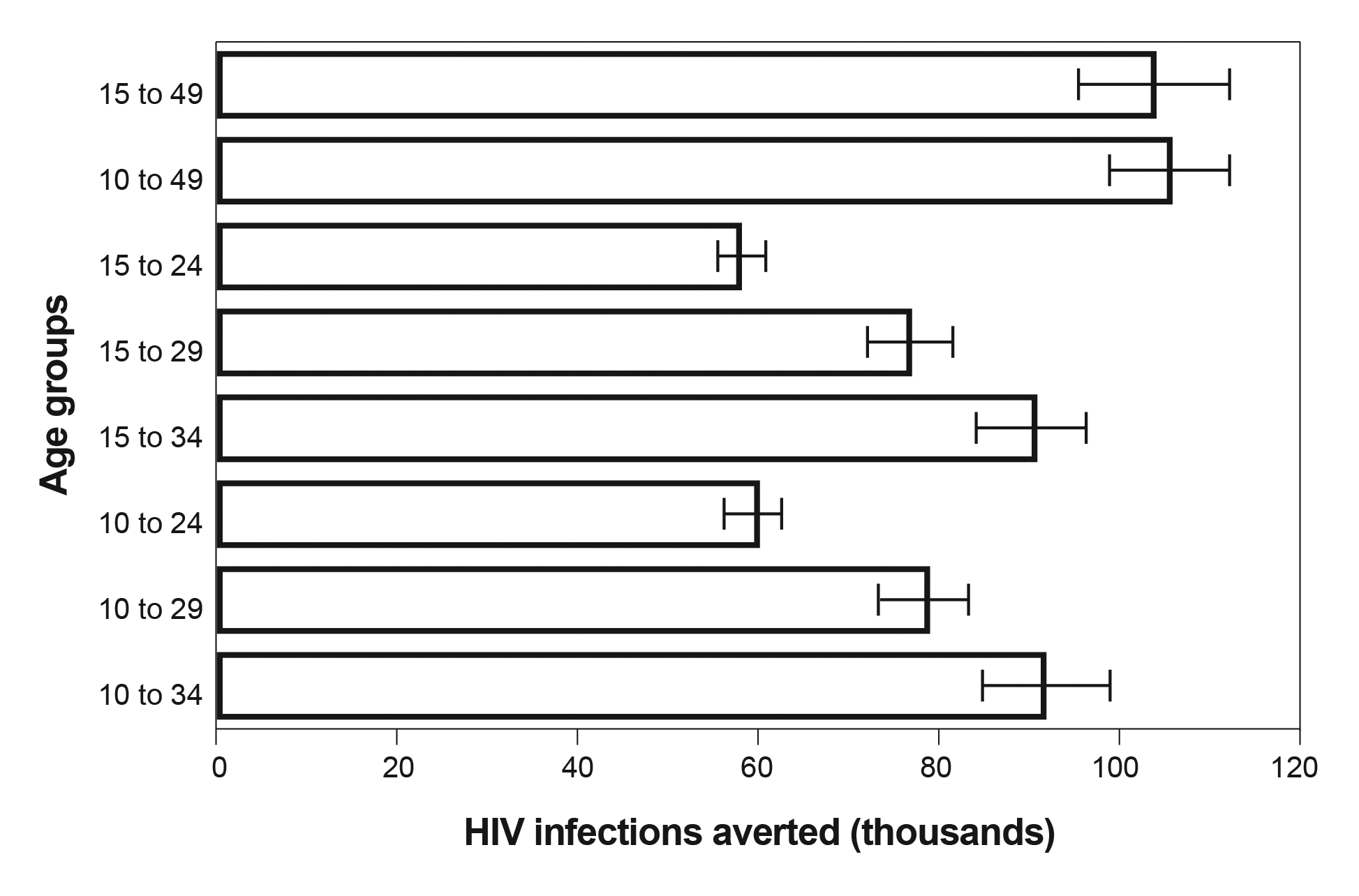

Supplement: S3 Fig — This figure illustrates the magnitude of impact, or the total number of HIV infections averted by each age strategy. In contrast with the previous figure, S3 Fig depicts more realistic scenarios, in which circumcisions are not limited to specific five-year age groups but are scaled up to 60% coverage across wider combined age groups. The most impactful scenario would be one in which most clients are circumcised: the 10- to 49-year-olds scenario. Error bars represent lower and upper uncertainty bounds as described in [17]. (TIF) [file pone.0156521.s003.tif]

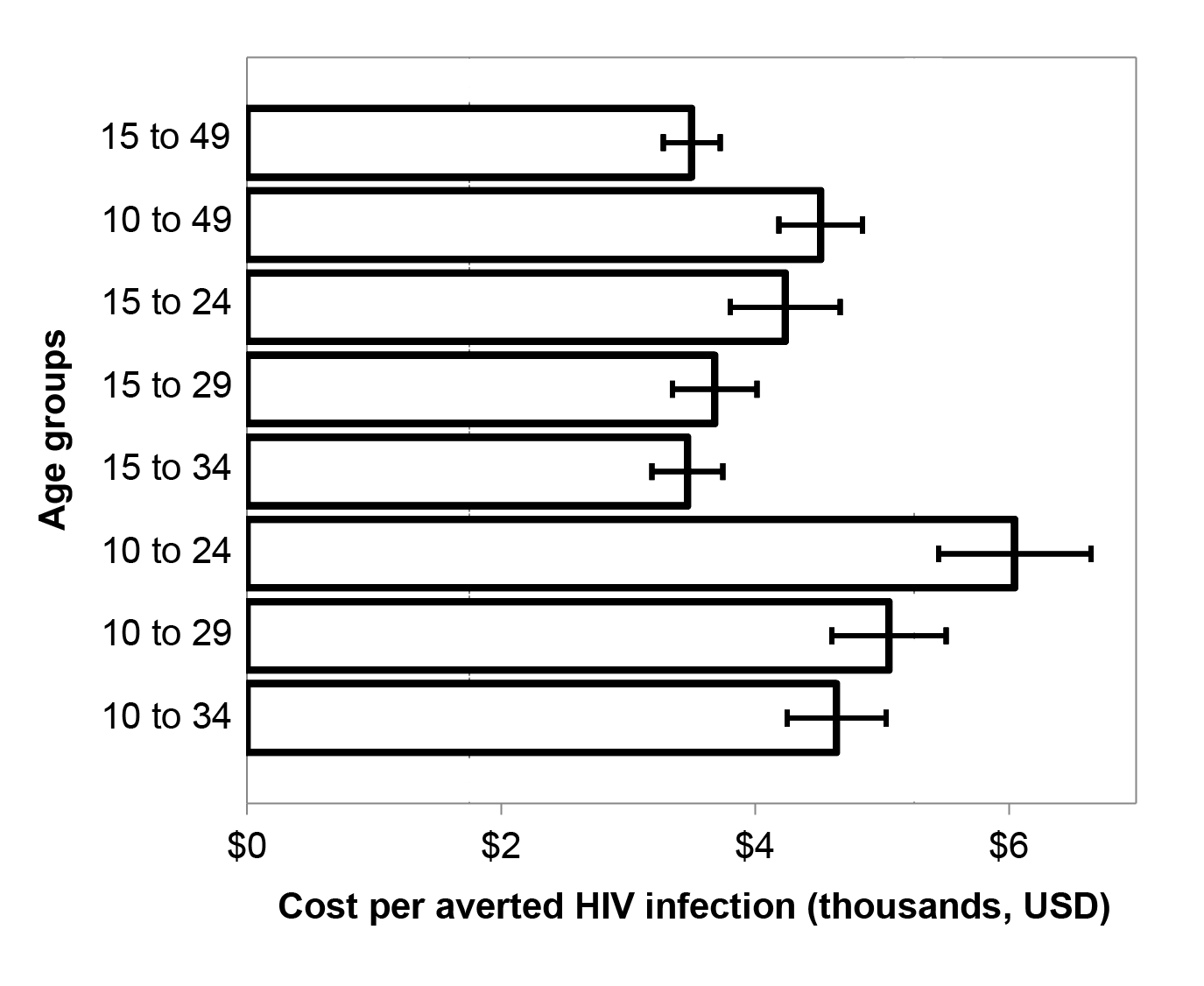

Supplement: S4 Fig — This figure compares the cost per HIV infection averted in each indicated scenario. As in S3 Fig, the scenarios compared here are combined age groups, and they again involve scaling up circumcision coverage to 60% of males in each indicated age group. Circumcising 60% of 15- to 49-year-olds would result in a cost of $3,500 per each infection averted. The lowest cost per HIV infection averted is achieved by circumcising males ages 15–34. Error bars represent lower and upper uncertainty bounds as described in [17]. (TIF) [file pone.0156521.s004.tif]
